# Ethyl 2-amino-4-(3-nitro­phen­yl)-4*H*-1-benzothieno[3,2-*b*]pyran-3-carboxyl­ate

**DOI:** 10.1107/S1600536814008538

**Published:** 2014-04-18

**Authors:** Mohamed Bakhouch, Ghali Al Houari, Mohamed El Yazidi, Mohamed Saadi, Lahcen El Ammari

**Affiliations:** a Département de Chimie, Faculté des Sciences, Dhar Mehraz, BP 1796 Atlas, 30000 Fes, Morocco; b Laboratoire de Chimie du Solide Appliquée, Faculté des Sciences, Université Mohammed V-Agdal, Avenue Ibn Battouta, BP 1014, Rabat, Morocco

## Abstract

The mol­ecule of the title compound, C_20_H_16_N_2_O_5_S, is built up by one fused five-membered and two fused six-membered rings linked to eth­oxy­carbonyl and 3-nitro­phenyl groups. The benzothieno­pyran ring system is nearly planar (r.m.s deviation = 0.0392 Å) and forms a dihedral angle of 86.90 (6)° with the aromatic ring of the nitro­benzene group. In the crystal, mol­ecules are linked by N—H⋯O hydrogen bonds and by π–π inter­actions between the phenyl ring and the six-membered heterocyle [inter­centroid distance = 3.5819 (8) Å], forming a three-dimensional network.

## Related literature   

For background to the organic synthesis of the title compound, see: House (1972 ▶); Kabashima *et al.* (2000 ▶); Jung (1991 ▶). For the preparation of heterocyclic compounds using condensation reactions, see: Boughaleb *et al.* (2011 ▶); Cabiddu *et al.* (2002 ▶); Pradhan & Asish (2005 ▶).
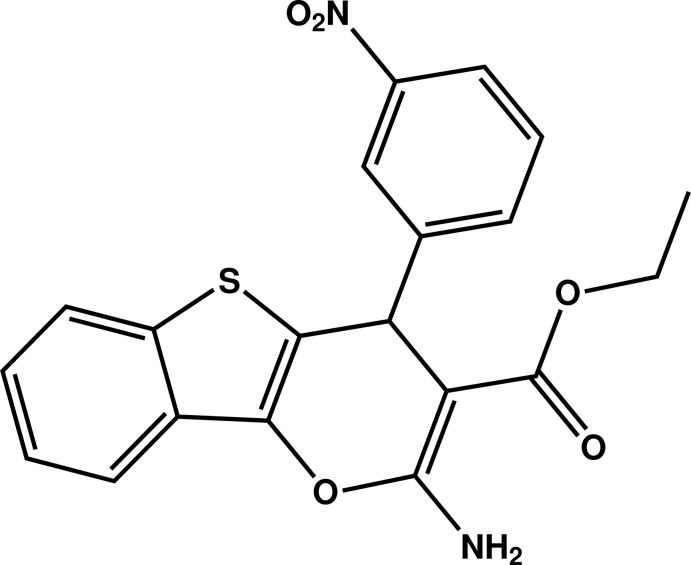


## Experimental   

### 

#### Crystal data  

C_20_H_16_N_2_O_5_S
*M* *_r_* = 396.41
Triclinic, 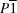

*a* = 8.3670 (2) Å
*b* = 9.4319 (2) Å
*c* = 12.8948 (4) Å
α = 102.505 (1)°
β = 106.493 (1)°
γ = 94.840 (1)°
*V* = 940.96 (4) Å^3^
*Z* = 2
Mo *K*α radiation
μ = 0.21 mm^−1^
*T* = 296 K
0.42 × 0.31 × 0.26 mm

#### Data collection  

Bruker X8 APEX diffractometer
Absorption correction: multi-scan (*SADABS*; Bruker, 2009 ▶) *T*_min_ = 0.673, *T*_max_ = 0.746
20668 measured reflections
4857 independent reflections
3954 reflections with *I* > 2σ(*I*)
*R*_int_ = 0.025

#### Refinement  

*R*[*F* ^2^ > 2σ(*F* ^2^)] = 0.044
*wR*(*F* ^2^) = 0.137
*S* = 1.05
4857 reflections
253 parameters
H-atom parameters constrained
Δρ_max_ = 0.37 e Å^−3^
Δρ_min_ = −0.25 e Å^−3^

### 

Data collection: *APEX2* (Bruker, 2009 ▶); cell refinement: *SAINT* (Bruker, 2009 ▶); data reduction: *SAINT*; program(s) used to solve structure: *SHELXS97* (Sheldrick, 2008 ▶); program(s) used to refine structure: *SHELXL97* (Sheldrick, 2008 ▶); molecular graphics: *ORTEP-3 for Windows* (Farrugia, 2012 ▶); software used to prepare material for publication: *PLATON* (Spek, 2009 ▶) and *publCIF* (Westrip, 2010 ▶).

## Supplementary Material

Crystal structure: contains datablock(s) I. DOI: 10.1107/S1600536814008538/rz5118sup1.cif

Structure factors: contains datablock(s) I. DOI: 10.1107/S1600536814008538/rz5118Isup2.hkl

Supporting information file. DOI: 10.1107/S1600536814008538/rz5118Isup3.cml

CCDC reference: 997474

Additional supporting information:  crystallographic information; 3D view; checkCIF report

## Figures and Tables

**Table 1:** Hydrogen-bond geometry (Å, °)

*D*—H⋯*A*	*D*—H	H⋯*A*	*D*⋯*A*	*D*—H⋯*A*
N1—H1*B*⋯O2	0.86	2.09	2.6950 (17)	127
N1—H1*B*⋯O2^i^	0.86	2.30	3.0327 (17)	143
N1—H1*A*⋯O5^ii^	0.86	2.30	3.1489 (19)	169

Symmetry codes: (i) 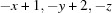; (ii) 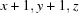.

**Fig. 1.**
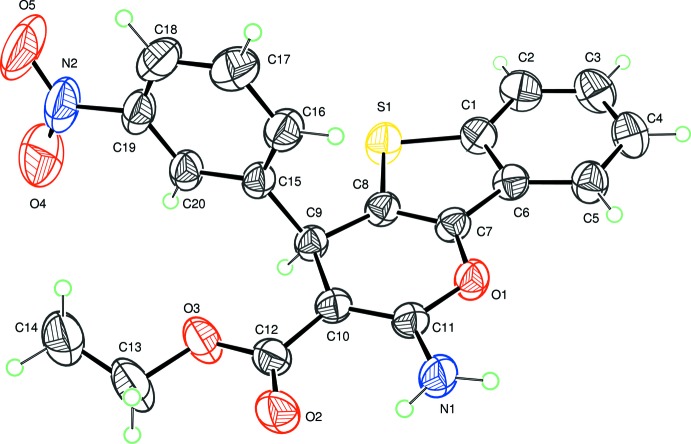
The molecular structure of the title compound with displacement ellipsoids drawn at the 50% probability level. H atoms are represented as small circles.

**Fig. 2.**
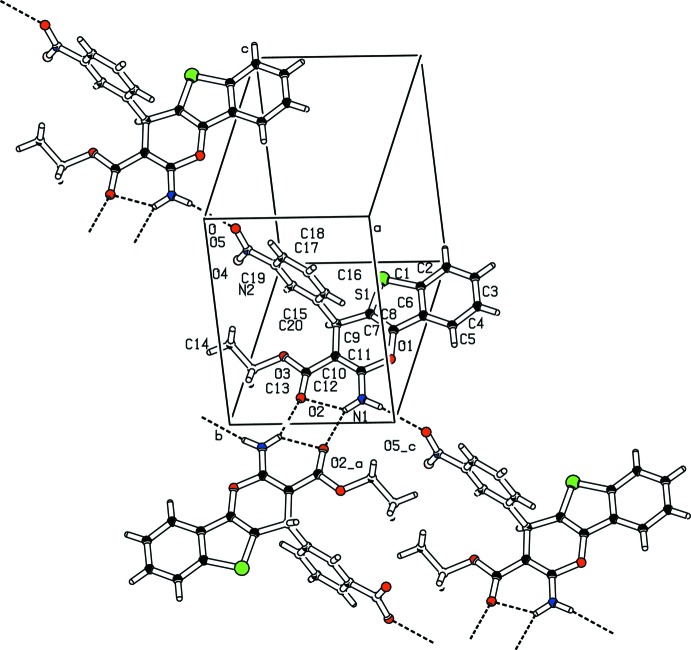
Partial crystal packing for the title compound showing molecules linked by hydrogen bonds (dashed lines).

## supplementary crystallographic information

### 1. Comment

The Michael reaction is one of the most efficient methods for effecting carbon–carbon bond formation and has wide synthetic applications (House, 1972; Kabashima *et al.*, 2000). This reaction and its close variants have been extensively used in organic synthesis (Jung, 1991). Generally, Michael additions are conducted in a suitable solvent in the presence of a strong base either at room or at elevated temperatures. In continuing our previous works on the preparation of hetrocyclic compounds by using condensation reactions (Boughaleb *et al.*, 2011), we now wish to describe the behavior of ethylcyanoacetate with respect to (*Z*)-2-(3-nitrobenzylidene)-1-benzo[*b*]thiophen-3(2*H*)-one and derivatives in ethanol, with the presence of piperidine as a basic catalyst (Cabiddu *et al.*, 2002; Pradhan & Asish, 2005). We have shown that cyclocondensation start with a Michael 1,4-additon, followed by intramolecular cyclization *via* nucleophilic addition of the hydroxyl group to the cyano group and not onto the carboxylate, to afford the tricyclic heterocycle ethyl2-amino-4-(3-nitrophenyl)-4*H*-1-benzothieno[3,2-*b*]pyran-3-carboxylate.

The molecule of the title compound, is formed by tree fused rings linked to an ethyl-3-carboxylate nd a 3-nitrophenyl group as shown in Fig. 1. The three fused rings (S1/C1–C11/O1) are almost coplanar, with the maximum deviation from the mean plane of -0.089 (2) Å at C9, and make a dihedral angle of 86.90 (6)° with the plane through the attached nitrophenyl group.

In the crystal, molecules are linked by N—H···O hydrogen bonds and by π–π interactions in a three-dimensional network as shown in Fig. 2 and Table 1.

### 2. Experimental

In a 100 ml flask equipped with a condenser was dissolved 4 mmol of (*Z*)-2-(3-nitrobenzylidene)-1-benzo[*b*]thiophen-3(2*H*)-one and 5 mmol of ethyl cyanoacetate in 30 ml of ethanol. Then, 1 ml of piperidine was added, and the reaction mixture was refluxed for 6 h. Thin layer chromatography revealed the formation of a single product. The organic phase was evaporated under reduce pressure. The resulting residue was recristallized from ethanol (Yield: 68%; m.p.: 493 K).

### 3. Refinement

H atoms were located in a difference map and treated as riding with C–H = 0.93–0.97 Å, N–H = 0.86 Å, and with *U*_iso_(H) = 1.2 *U*_eq_(C, N) or *U*_iso_(H) = 1.5 *U*_eq_(C) for methyl H atoms. Two ouliers (0 0 1, 0 1 0) were omitted in the last cycles of refinement.

### Crystal data

**Table:**

C_20_H_16_N_2_O_5_S	*Z* = 2
*M**_r_* = 396.41	*F*(000) = 412
Triclinic, *P*1	*D*_x_ = 1.399 Mg m^−^^3^
Hall symbol: -P 1	Melting point: 493 K
*a* = 8.3670 (2) Å	Mo *K*α radiation, λ = 0.71073 Å
*b* = 9.4319 (2) Å	Cell parameters from 4857 reflections
*c* = 12.8948 (4) Å	θ = 2.5–28.7°
α = 102.505 (1)°	µ = 0.21 mm^−^^1^
β = 106.493 (1)°	*T* = 296 K
γ = 94.840 (1)°	Block, colourless
*V* = 940.96 (4) Å^3^	0.42 × 0.31 × 0.26 mm

### Data collection

**Table:**

Bruker X8 APEX diffractometer	4857 independent reflections
Radiation source: fine-focus sealed tube	3954 reflections with *I* > 2σ(*I*)
Graphite monochromator	*R*_int_ = 0.025
φ and ω scans	θ_max_ = 28.7°, θ_min_ = 2.5°
Absorption correction: multi-scan (*SADABS*; Bruker, 2009)	*h* = −11→11
*T*_min_ = 0.673, *T*_max_ = 0.746	*k* = −12→12
20668 measured reflections	*l* = −17→17

### Refinement

**Table:**

Refinement on *F*^2^	Primary atom site location: structure-invariant direct methods
Least-squares matrix: full	Secondary atom site location: difference Fourier map
*R*[*F*^2^ > 2σ(*F*^2^)] = 0.044	Hydrogen site location: inferred from neighbouring sites
*wR*(*F*^2^) = 0.137	H-atom parameters constrained
*S* = 1.05	*w* = 1/[σ^2^(*F*_o_^2^) + (0.0782*P*)^2^ + 0.1615*P*] where *P* = (*F*_o_^2^ + 2*F*_c_^2^)/3
4857 reflections	(Δ/σ)_max_ = 0.001
253 parameters	Δρ_max_ = 0.37 e Å^−^^3^
0 restraints	Δρ_min_ = −0.25 e Å^−^^3^

### Special details

**Table:**

Geometry. All e.s.d.'s (except the e.s.d. in the dihedral angle between two l.s. planes) are estimated using the full covariance matrix. The cell e.s.d.'s are taken into account individually in the estimation of e.s.d.'s in distances, angles and torsion angles; correlations between e.s.d.'s in cell parameters are only used when they are defined by crystal symmetry. An approximate (isotropic) treatment of cell e.s.d.'s is used for estimating e.s.d.'s involving l.s. planes.
Refinement. Refinement of *F*^2^ against all reflections. The weighted *R*-factor *wR* and goodness of fit *S* are based on *F*^2^, conventional *R*-factors *R* are based on *F*, with *F* set to zero for negative *F*^2^. The threshold expression of *F*^2^ > σ(*F*^2^) is used only for calculating *R*-factors(gt) *etc*. and is not relevant to the choice of reflections for refinement. *R*-factors based on *F*^2^ are statistically about twice as large as those based on *F*, and *R*- factors based on all data will be even larger.

### Fractional atomic coordinates and isotropic or equivalent isotropic displacement parameters (Å^2^)

**Table:**

	*x*	*y*	*z*	*U*_iso_*/*U*_eq_	
C1	1.03581 (19)	0.74569 (16)	0.51586 (12)	0.0444 (3)	
C2	1.1714 (2)	0.7184 (2)	0.59752 (14)	0.0572 (4)	
H2	1.1539	0.6646	0.6469	0.069*	
C3	1.3318 (2)	0.7739 (2)	0.60256 (14)	0.0596 (4)	
H3	1.4239	0.7566	0.6561	0.072*	
C4	1.3590 (2)	0.8552 (2)	0.52938 (14)	0.0553 (4)	
H4	1.4689	0.8908	0.5347	0.066*	
C5	1.22638 (19)	0.88383 (17)	0.44927 (13)	0.0472 (3)	
H5	1.2455	0.9388	0.4010	0.057*	
C6	1.06239 (17)	0.82841 (14)	0.44214 (11)	0.0388 (3)	
C7	0.90329 (17)	0.83869 (14)	0.36665 (11)	0.0366 (3)	
C8	0.76677 (17)	0.76734 (14)	0.37893 (11)	0.0373 (3)	
C9	0.59063 (16)	0.75463 (14)	0.30354 (11)	0.0356 (3)	
H9	0.5205	0.7943	0.3487	0.043*	
C10	0.59569 (16)	0.84817 (14)	0.22130 (11)	0.0361 (3)	
C11	0.74244 (17)	0.91953 (14)	0.21693 (11)	0.0371 (3)	
C12	0.43789 (18)	0.86851 (15)	0.14814 (12)	0.0418 (3)	
C13	0.1380 (2)	0.8102 (3)	0.0990 (2)	0.0859 (7)	
H13A	0.1389	0.8011	0.0228	0.103*	
H13B	0.1031	0.9034	0.1248	0.103*	
C14	0.0200 (2)	0.6887 (2)	0.1031 (2)	0.0785 (6)	
H14A	−0.0914	0.6910	0.0564	0.118*	
H14B	0.0192	0.6988	0.1787	0.118*	
H14C	0.0549	0.5969	0.0770	0.118*	
C15	0.51766 (16)	0.59335 (14)	0.24462 (11)	0.0363 (3)	
C16	0.5980 (2)	0.50862 (16)	0.17907 (13)	0.0486 (3)	
H16	0.6969	0.5507	0.1710	0.058*	
C17	0.5336 (2)	0.36266 (18)	0.12555 (16)	0.0598 (4)	
H17	0.5895	0.3077	0.0820	0.072*	
C18	0.3863 (2)	0.29794 (17)	0.13649 (15)	0.0592 (5)	
H18	0.3414	0.2000	0.1008	0.071*	
C19	0.30905 (19)	0.38353 (17)	0.20176 (14)	0.0516 (4)	
C20	0.37110 (17)	0.52953 (16)	0.25679 (12)	0.0439 (3)	
H20	0.3155	0.5835	0.3010	0.053*	
N1	0.75846 (17)	0.99963 (14)	0.14576 (11)	0.0500 (3)	
H1A	0.8569	1.0394	0.1494	0.060*	
H1B	0.6704	1.0114	0.0963	0.060*	
N2	0.1493 (2)	0.3189 (2)	0.21188 (16)	0.0745 (5)	
O1	0.89774 (12)	0.91629 (11)	0.28705 (8)	0.0435 (2)	
O2	0.41926 (14)	0.93260 (13)	0.07393 (10)	0.0551 (3)	
O3	0.30471 (13)	0.80479 (15)	0.16981 (11)	0.0597 (3)	
O4	0.0661 (2)	0.3993 (2)	0.2526 (2)	0.1098 (7)	
O5	0.1064 (2)	0.1868 (2)	0.17700 (19)	0.1165 (7)	
S1	0.82193 (5)	0.68473 (5)	0.48947 (3)	0.05225 (14)	

### Atomic displacement parameters (Å^2^)

**Table:**

	*U*^11^	*U*^22^	*U*^33^	*U*^12^	*U*^13^	*U*^23^
C1	0.0450 (7)	0.0467 (7)	0.0397 (7)	0.0057 (6)	0.0084 (6)	0.0138 (6)
C2	0.0577 (10)	0.0676 (10)	0.0468 (8)	0.0126 (8)	0.0067 (7)	0.0273 (7)
C3	0.0479 (9)	0.0773 (11)	0.0482 (9)	0.0150 (8)	0.0012 (7)	0.0211 (8)
C4	0.0391 (8)	0.0712 (10)	0.0487 (9)	0.0056 (7)	0.0063 (6)	0.0119 (7)
C5	0.0410 (7)	0.0539 (8)	0.0423 (7)	0.0017 (6)	0.0072 (6)	0.0130 (6)
C6	0.0404 (7)	0.0371 (6)	0.0342 (6)	0.0037 (5)	0.0068 (5)	0.0064 (5)
C7	0.0392 (7)	0.0346 (6)	0.0343 (6)	0.0025 (5)	0.0082 (5)	0.0107 (5)
C8	0.0394 (7)	0.0373 (6)	0.0343 (6)	0.0030 (5)	0.0083 (5)	0.0126 (5)
C9	0.0349 (6)	0.0380 (6)	0.0360 (6)	0.0032 (5)	0.0121 (5)	0.0129 (5)
C10	0.0377 (7)	0.0355 (6)	0.0373 (6)	0.0054 (5)	0.0113 (5)	0.0139 (5)
C11	0.0380 (6)	0.0356 (6)	0.0386 (7)	0.0054 (5)	0.0106 (5)	0.0131 (5)
C12	0.0388 (7)	0.0452 (7)	0.0477 (8)	0.0116 (5)	0.0156 (6)	0.0197 (6)
C13	0.0361 (9)	0.1282 (19)	0.1168 (18)	0.0216 (10)	0.0176 (10)	0.0839 (16)
C14	0.0483 (10)	0.0803 (13)	0.0881 (15)	0.0057 (9)	0.0018 (10)	0.0096 (11)
C15	0.0341 (6)	0.0387 (6)	0.0365 (6)	0.0011 (5)	0.0075 (5)	0.0166 (5)
C16	0.0476 (8)	0.0441 (7)	0.0552 (9)	0.0014 (6)	0.0198 (7)	0.0119 (6)
C17	0.0684 (11)	0.0455 (8)	0.0610 (10)	0.0067 (7)	0.0188 (9)	0.0067 (7)
C18	0.0655 (10)	0.0412 (7)	0.0560 (9)	−0.0078 (7)	−0.0024 (8)	0.0160 (7)
C19	0.0409 (7)	0.0545 (8)	0.0533 (8)	−0.0106 (6)	−0.0014 (6)	0.0292 (7)
C20	0.0358 (7)	0.0509 (7)	0.0467 (8)	−0.0001 (6)	0.0094 (6)	0.0228 (6)
N1	0.0416 (6)	0.0573 (7)	0.0569 (8)	0.0010 (5)	0.0113 (6)	0.0342 (6)
N2	0.0517 (9)	0.0814 (11)	0.0832 (11)	−0.0221 (8)	0.0030 (8)	0.0424 (9)
O1	0.0356 (5)	0.0502 (5)	0.0465 (5)	−0.0001 (4)	0.0085 (4)	0.0239 (4)
O2	0.0458 (6)	0.0699 (7)	0.0608 (7)	0.0148 (5)	0.0145 (5)	0.0404 (6)
O3	0.0343 (5)	0.0866 (8)	0.0737 (8)	0.0155 (5)	0.0171 (5)	0.0496 (7)
O4	0.0650 (10)	0.1204 (15)	0.1621 (19)	−0.0096 (10)	0.0533 (12)	0.0581 (14)
O5	0.0925 (12)	0.0841 (11)	0.1571 (18)	−0.0447 (9)	0.0255 (12)	0.0378 (11)
S1	0.0472 (2)	0.0633 (3)	0.0485 (2)	−0.00050 (17)	0.00801 (16)	0.03121 (18)

### Geometric parameters (Å, º)

**Table:**

C1—C2	1.396 (2)	C12—O3	1.3482 (17)
C1—C6	1.405 (2)	C13—O3	1.446 (2)
C1—S1	1.7449 (16)	C13—C14	1.469 (3)
C2—C3	1.377 (3)	C13—H13A	0.9700
C2—H2	0.9300	C13—H13B	0.9700
C3—C4	1.392 (3)	C14—H14A	0.9600
C3—H3	0.9300	C14—H14B	0.9600
C4—C5	1.376 (2)	C14—H14C	0.9600
C4—H4	0.9300	C15—C20	1.3843 (18)
C5—C6	1.397 (2)	C15—C16	1.388 (2)
C5—H5	0.9300	C16—C17	1.383 (2)
C6—C7	1.4338 (18)	C16—H16	0.9300
C7—C8	1.3423 (18)	C17—C18	1.386 (3)
C7—O1	1.3768 (15)	C17—H17	0.9300
C8—C9	1.4959 (18)	C18—C19	1.371 (3)
C8—S1	1.7378 (14)	C18—H18	0.9300
C9—C10	1.5261 (17)	C19—C20	1.384 (2)
C9—C15	1.5308 (17)	C19—N2	1.473 (2)
C9—H9	0.9800	C20—H20	0.9300
C10—C11	1.3712 (18)	N1—H1A	0.8600
C10—C12	1.4436 (19)	N1—H1B	0.8600
C11—N1	1.3356 (17)	N2—O4	1.208 (3)
C11—O1	1.3633 (16)	N2—O5	1.214 (2)
C12—O2	1.2195 (17)		
			
C2—C1—C6	120.99 (15)	O3—C13—C14	108.58 (16)
C2—C1—S1	127.04 (13)	O3—C13—H13A	110.0
C6—C1—S1	111.96 (11)	C14—C13—H13A	110.0
C3—C2—C1	117.84 (16)	O3—C13—H13B	110.0
C3—C2—H2	121.1	C14—C13—H13B	110.0
C1—C2—H2	121.1	H13A—C13—H13B	108.4
C2—C3—C4	121.46 (15)	C13—C14—H14A	109.5
C2—C3—H3	119.3	C13—C14—H14B	109.5
C4—C3—H3	119.3	H14A—C14—H14B	109.5
C5—C4—C3	121.21 (16)	C13—C14—H14C	109.5
C5—C4—H4	119.4	H14A—C14—H14C	109.5
C3—C4—H4	119.4	H14B—C14—H14C	109.5
C4—C5—C6	118.47 (15)	C20—C15—C16	119.01 (13)
C4—C5—H5	120.8	C20—C15—C9	120.39 (12)
C6—C5—H5	120.8	C16—C15—C9	120.59 (11)
C5—C6—C1	120.03 (13)	C17—C16—C15	121.20 (15)
C5—C6—C7	130.31 (13)	C17—C16—H16	119.4
C1—C6—C7	109.65 (12)	C15—C16—H16	119.4
C8—C7—O1	124.23 (12)	C16—C17—C18	120.27 (17)
C8—C7—C6	115.68 (12)	C16—C17—H17	119.9
O1—C7—C6	120.09 (11)	C18—C17—H17	119.9
C7—C8—C9	124.19 (12)	C19—C18—C17	117.59 (14)
C7—C8—S1	111.37 (10)	C19—C18—H18	121.2
C9—C8—S1	124.38 (10)	C17—C18—H18	121.2
C8—C9—C10	107.83 (10)	C18—C19—C20	123.38 (14)
C8—C9—C15	110.43 (11)	C18—C19—N2	118.73 (15)
C10—C9—C15	112.40 (10)	C20—C19—N2	117.87 (17)
C8—C9—H9	108.7	C19—C20—C15	118.54 (15)
C10—C9—H9	108.7	C19—C20—H20	120.7
C15—C9—H9	108.7	C15—C20—H20	120.7
C11—C10—C12	118.22 (12)	C11—N1—H1A	120.0
C11—C10—C9	123.25 (12)	C11—N1—H1B	120.0
C12—C10—C9	118.45 (11)	H1A—N1—H1B	120.0
N1—C11—O1	109.61 (11)	O4—N2—O5	123.09 (19)
N1—C11—C10	127.07 (13)	O4—N2—C19	118.92 (16)
O1—C11—C10	123.32 (12)	O5—N2—C19	118.0 (2)
O2—C12—O3	121.52 (13)	C11—O1—C7	116.95 (10)
O2—C12—C10	126.91 (13)	C12—O3—C13	117.57 (13)
O3—C12—C10	111.56 (12)	C8—S1—C1	91.31 (7)

### Hydrogen-bond geometry (Å, º)

**Table:**

*D*—H···*A*	*D*—H	H···*A*	*D*···*A*	*D*—H···*A*
N1—H1*B*···O2	0.86	2.09	2.6950 (17)	127
N1—H1*B*···O2^i^	0.86	2.30	3.0327 (17)	143
N1—H1*A*···O5^ii^	0.86	2.30	3.1489 (19)	169

Symmetry codes: (i) −*x*+1, −*y*+2, −*z*; (ii) *x*+1, *y*+1, *z*.

## References

[bb1] Boughaleb, A., Zouihri, H., Gmouh, S., Kerbal, A. & El yazidi, M. (2011). *Acta Cryst.* E**67**, o2106. 10.1107/S1600536811027152 PMC3213548 22091125

[bb2] Bruker (2009). *APEX2*, *SAINT* and *SADABS* Bruker AXS Inc., Madison, Wisconsin, USA.

[bb3] Cabiddu, M. G., Cabiddu, S., Cadoni, E., De Montis, S., Fattuoni, C., Melis, S. & Usai, M. (2002). *Synthesis*, **7**, 875–878.

[bb4] Farrugia, L. J. (2012). *J. Appl. Cryst.* **45**, 849–854.

[bb5] House, H. O. (1972). *Modern Synthetic Reactions*, 2nd ed., pp. 581–595. New York: Benjamin.

[bb6] Jung, M. E. (1991). *Comprehensive Organic Synthesis*, Vol. 4, edited by B. M. Trost, I. Fleming & M. F. Semmelhack, pp. 1–68. Oxford: Pergamon Press.

[bb7] Kabashima, H., Tsuji, H., Shibuya, T. & Hattori, H. (2000). *J. Mol. Catal. A Chem.* **155**, 23–29.

[bb8] Pradhan, T. K. & Asish, D. (2005). *Tetrahedron Lett.* **61**, 9007–9017.

[bb9] Sheldrick, G. M. (2008). *Acta Cryst.* A**64**, 112–122. 10.1107/S0108767307043930 18156677

[bb10] Spek, A. L. (2009). *Acta Cryst.* D**65**, 148–155. 10.1107/S090744490804362X PMC2631630 19171970

[bb11] Westrip, S. P. (2010). *J. Appl. Cryst.* **43**, 920–925.

